# Modulatory Effect of Cattle on Risk for Lyme Disease

**DOI:** 10.3201/eid1212.051552

**Published:** 2006-12

**Authors:** Dania Richter, Franz-Rainer Matuschka

**Affiliations:** * Charité Universitätsmedizin Berlin, Berlin, Germany

**Keywords:** Lyme disease, prevention, zooprophylaxis, landscape management, research

## Abstract

To determine the effect of cattle on the risk for Lyme disease, we compared the prevalence of spirochete infection in questing vector ticks collected from a pasture with low-intensity cattle grazing with the prevalence in those collected from a site on which no cattle grazed. The presence of cattle limited the prevalence of *Borrelia burgdorferi* s.l., but not *B. miyamotoi*, in vector ticks. The reintroduction of traditional, nonintensive agriculture in central Europe may help reduce risk for Lyme disease.

## Abstract 2

TOC Summary: Low-intensity cattle grazing limits *Borrelia burgdorferi* s.l., but not *B. miyamotoi,* in vector ticks.

The tick Ixodes ricinus, which transmits the spirochete agent of Lyme disease, is abundant in the ecotone at a forest's edge. The brushy vegetation there provides a suitable microclimate for these vector ticks as well as food and cover for the animals that serve as hosts for the ticks' subadult stages. Of those, rodents and particular passerine birds serve as reservoirs for the agent of Lyme disease (*1**–**8*). Other animals, such as domestic ungulates, serve as hosts for the tick but fail to support the pathogen. These animals exert a zooprophylactic effect on the transmission cycle by diverting ticks from feeding on reservoir-competent hosts. Any tick feeding on these hosts fails to acquire spirochetes and, thereby, fails to contribute to transmission (*9**,**10*). In addition, infected ticks may lose the pathogen when feeding on such incompetent hosts (*11**–**14*). The ecotone constitutes the focus of transmission, and persons may acquire numerous vector ticks when passing through such a site. The local composition of vertebrate animals serving as hosts to vector ticks determines the risk for infection at that site.

Recently, the European Union and the United Nations Educational, Scientific and Cultural Organization (UNESCO) instituted programs to limit the growth of brush and subsequent reforestation of uncultivated land. These programs specifically target the ecotonal habitat that developed ubiquitously in Europe when cultivating marginal land was considered too labor-intensive and costly. Toward this end, traditional farming methods are being reimplemented. Domestic ungulates, for example, are being introduced into the ecotone for landscape management. By grazing or browsing on scrub brush and saplings, these animals help maintain open landscapes and simultaneously enhance biodiversity. Although domestic animals may alter the contours of the ecotone, the effect of their presence on the risk for human infection by the agent of Lyme disease has not been defined.

To determine whether the presence of domestic ungulates may reduce risk for human Lyme disease, we compared the prevalence of spirochetal infection in ticks taken from an ecotonal cattle pasture to that in a site in which no cattle were permitted to graze. In addition, we determined the prevalence of the different Lyme disease genospecies in these ticks and that of a distantly related spirochete.

## Materials and Methods

The study site, ≈2 km^2^, is located near the town of Lembach, in the northern Vosges region of France, at the floodplain of the Sauer River. It is part of the Pfälzer Wald–Vosges du Nord biosphere reserve of UNESCO. Scottish Highland cattle were introduced there in 1999 for low-intensity grazing. About 250 cattle are allowed to graze in the floodplain year-round and are rotated on grazing patches enclosed by electric fencing on a total area of ≈200 km^2^. The fence does not constitute a barrier to other vertebrates, such as hedgehogs, foxes, or deer.

On the cattle pasture and at a site 10 m outside the pasture, questing ticks were sampled by passing a flannel flag over the vegetation; ticks were preserved in 80% ethanol. We sampled the sites twice, in early May and early June 2005. Ticks were collected from 3 distinct areas within and 2 areas outside the pasture site. Both sites are separated by a path ≈4 m wide. Microscopy was used to identify stage and species of all ticks.

To detect and identify the various spirochetes that may be present in these ticks, the opisthosoma of each was opened, the contained mass of soft tissue was dissected out and placed in physiologic saline, and DNA was extracted as described previously (*15*). Borrelia genospecies were detected and characterized by amplifying and sequencing a 600-nt fragment of the gene encoding the 16S rRNA by nested PCR (*15*). Each resulting sequence was compared with sequences of the homologous gene fragment representing each of the validated spirochete genospecies. The following sequences served for comparison: GenBank accession nos. X85196 and X85203 for B. burgdorferi s.s.; X85190, X85192, and X85194 for B. afzelii; X85193, X85199, and M64311 for B. garinii; X98228 and X98229 for B. lusitaniae; X98232 and X98233 for B. valaisiana; AY147008 for B. spielmanii; and AY253149 for B. miyamotoi. A complete match, permitting no more than 2 nt changes, was required.

## Results

To determine the effect of the presence of cattle on the prevalence of spirochetal infection in vector ticks, we sampled questing ticks within a fenced site where cattle grazed, examined them individually for spirochetal DNA by PCR and compared the resulting prevalence to that in ticks sampled from at least 10 m beyond the fence. Spirochetes infected ≈4× as many nymphs and ≈6× as many adults outside the enclosure as within (Table) (Fisher exact test, p<0.0001 for either developmental stage). The variance between different samples taken within the pasture site and the variance between samples taken outside the pasture were considerably smaller than the variance between the pasture and the nonpasture site. Fewer questing ticks harbored spirochetes where cattle were present than where they were absent.

**Table:** Prevalence of spirochetes in questing nymphal and adult *Ixodes ricinus* ticks sampled in and outside a cattle pasture, France*

Cattle in site	Ticks examined				% Ticks infected by *Borrelia*						
	Stage	No.	% Infected	±SD	*B. afzeli*	*B. garinii*	*B. valaisiana*	*B. burgdorferi s.s.*	*B. lusitaniae*	*B. spielmanii*	*B. miyamotoi*
Present	Nymph	238	5.5	5.8	2.9	0.4	0	0.4	0.4	0	1.3
	Adult	136	5.9	3.7	3.7†	1.5	0	0	0.7	0	0.7
Absent	Nymph	95	22.1	5.8	5.3	9.5	4.2†	0	4.2	0	0
	Adult	236	40.3	2.3	13.6†	12.7	3.0‡	0.4	7.6	1.3	3.0

*SD, standard deviation. †Includes a tick coinfected with *B. garinii*. ‡Includes 2 ticks coinfected with *B. garinii*.

We then determined whether the presence of cattle affects a spirochetal genospecies more than others. Amplified spirochetal DNA derived from each infected tick was sequenced and the genospecies determined by comparison with published sequences. Where cattle were present, virtually no nymphal or adult ticks were infected by B. valaisiana (Fisher exact test, p = 0.006, p = 0.05, respectively; Table). The prevalence of B. lusitaniae in ticks sampled on the cattle pasture was about one tenth the prevalence in ticks sampled outside the pasture (Fisher exact test, p = 0.025 in nymphs, p = 0.0025 in adults). B. garinii infected about one twentieth as many nymphal ticks and about one eighth as many adult ticks where cattle were present compared with the nonpasture site (Fisher exact test, p<0.0001 for either developmental stage). Whereas the prevalence of B. afzelii in nymphal ticks appeared to be not significantly affected by the presence of cattle, its prevalence in adult ticks was reduced to approximately one quarter (Fisher exact test, p = 0.0019). Too few ticks infected by B. burgdorferi s.s. or B. spielmanii were sampled to permit comparison. In contrast to the prevalence of Lyme disease spirochetes, the prevalence of B. miyamotoi, an ixodid-borne member of relapsing fever spirochetes, was similar, regardless of the presence of cattle. We conclude that the presence of cattle appears to reduce the prevalence of Lyme disease spirochetes in vector ticks but appears not to influence that of a distantly related spirochete.

## Discussion

A vertebrate animal originally was said to be zooprophylactic if its presence in an endemic site serves to divert invertebrate vectors of a human pathogen from feeding on people (*16*). The concept was extended for Lyme disease to include more specific diversionary effects on the force of transmission of the pathogen, including diversion of the vector tick from feeding on potential reservoir hosts, transportation of vector ticks away from the venue of transmission, or elimination of infection from a previously infected tick (*11*). Lizards, for example, fail to support Lyme disease spirochetes, and the prevalence of infection in questing vector ticks appears reduced where such hosts are abundant (*2**,**9*). Ticks may detach from birds in nonsuitable habitats. Zooprophylaxis may affect the prevalence of infection in vector ticks by various factors.

Wild ungulates are generally thought to contribute to risk for Lyme disease, mainly by supporting the population of vector ticks. Indeed, adult I. dammini (or scapularis) ticks in the northeastern United States appear to preferably feed on white-tailed deer (*17*). Because they serve as definitive host for the tick, density of deer correlates with tick abundance (*18**,**19*). Reduction or elimination of deer on islands or their exclusion by electric fences has resulted in fewer subadult ticks questing in subsequent years; the density of adult ticks, however, has declined only slowly (*20**–**23*). In contrast, a simulation model indicates that the density of ticks may be more sensitive to the availability of hosts for the subadult stages than to that of hosts for adult ticks (*24*). In Europe, diverse medium-sized and large mammals, such as hares, hedgehogs, and foxes, constitute alternative hosts for adult ticks (*25**–**27*). As a result, any association between the abundance of vector ticks and the density of deer may be less profound in Europe than in eastern North America.

Domestic as well as wild ungulates exert a powerful zooprophylactic effect on Lyme disease spirochetes because they are incompetent for these pathogens. Not only do noninfected ticks that feed on these mammals fail to acquire spirochetes, but also infected ticks lose their infection during the course of such a bloodmeal (*10**,**12**,**28**,**29*). The sensitivity to the bacteriolytic activity of the complement pathway may determine the incompetence of a host (*30*). Cattle support the feeding of numerous ticks of all stages (*10*) and thereby divert subadult vector ticks from feeding on reservoir-competent hosts. A simulation model indicated that the availability of incompetent hosts for subadult tick stages would reduce prevalence of infection (*24*). Our observation that questing nymphal and adult ticks collected on a cattle pasture were less likely to be infected by Lyme disease spirochetes than were vector ticks collected outside the pasture supports this model. In addition, cattle and other domestic ungulates may modify the ecotonal vegetation by their grazing. This would render the microclimate more arid and, thereby, less suitable for the survival of ticks. Indeed, during equal time periods, we collected approximately half as many questing ticks where cattle were present as we did where cattle were absent (1 tick every 4 minutes vs. 2 minutes, respectively). Grazing cattle may also deplete reservoir-competent rodents and birds foraging in this ecotone of their food source and of cover from predators. By reducing the availability of potential reservoir hosts, additional subadult stages are diverted to feed on incompetent domestic ungulates. Whether the effect of cattle on the prevalence of spirochete infection in questing vector ticks results from the inability of cattle to support the pathogen or from their effect on tick habitat and reservoir rodents remains to be determined. In addition to their parasitologic effects, certain zooprophylactic animals may modify the ecotonal landscape in ways that affect transmission of the agent of Lyme disease.

Although the presence of cattle appears to suppress the prevalence of the various genospecies of Lyme disease spirochetes, that of B. miyamotoi is not affected. It is more closely related to spirochetes that cause relapsing fever than to spirochetes that cause Lyme disease (*31*). No disease relationship has yet been demonstrated for this microbe. B. miyamotoi spirochetes infect ≈2%–5% of questing nymphal or adult I. ricinus–like ticks, regardless of location (*15**,**32*). The prevalence of Lyme disease spirochetes, on the other hand, varies from zero to ≈50%, depending on the local composition of reservoir populations. Our observation that the prevalence of B. miyamotoi is insensitive to the presence of cattle corroborates reports on transovarial transmission (*32*) and suggests that this spirochete may cycle solely within the vector population.

The European Union and UNESCO support various policies for the deintensification of agriculture. The European Union's Biodiversity Action Plan for Agriculture, for example, recognizes that traditional nonintensive agriculture, as it was practiced for centuries until only a few decades ago, maintained an enormous species variety in wild and domestic flora and fauna (*33*). By encouraging extensive agricultural practices, such programs aim to prevent further abandonment of marginal farmland and the subsequent development of scrub brush and forests that support relatively few species. Management of previous fallow land as low-intensity pastures for robust varieties of cattle, sheep, goats, horses, or pigs helps maintain or reestablish not only open landscapes but also high nature value habitats (high biodiversity achieved by extensive farming). Our finding that the prevalence of Lyme disease spirochetes in questing vector ticks is significantly reduced on such pastures suggests that enhanced biodiversity may also contribute directly to human health. Conclusions derived from a mathematical model similarly suggest that a species-rich composition of vertebrates that serve as hosts for vector ticks would decrease the prevalence of spirochete-infected ticks by diluting the effect of reservoir-competent hosts (*34*). Efforts aimed at extensive landscape management may help to reduce risk for Lyme disease in central Europe.

We conclude that cattle appear to modulate risk for Lyme disease by reducing the prevalence of Lyme disease spirochetes in vector ticks; they do not, however, influence prevalence of a distantly related spirochete. Our observations were conducted in a high nature-value habitat that included an extensively managed cattle pasture. In addition to the direct parasitologic effects, certain zooprophylactic animals may modify the landscape in ways that affect the transmission of Lyme disease spirochetes. Removal of ground-covering vegetation may reduce the force of transmission of this pathogen by reducing local rodent density, thereby diverting vector ticks to reservoir-incompetent cattle. Zooprophylaxis may include environmental modification. Our finding that the prevalence of B. miyamotoi is insensitive to the presence of cattle suggests that this spirochete may be perpetuated differently than are Lyme disease spirochetes. Extensive landscape management in central Europe may help reduce risk for Lyme disease.
